# DPP4 inhibitors as a novel therapeutic strategy in colorectal cancer: Integrating network biology and experimental insights

**DOI:** 10.1371/journal.pone.0334223

**Published:** 2025-10-10

**Authors:** Sanaa K. Bardaweel, Baraa Abu Sneineh, Rima Hajjo, Reema Abu Khalaf

**Affiliations:** 1 Department of Pharmaceutical Sciences, School of Pharmacy, University of Jordan, Amman, Jordan; 2 Department of Pharmacy, Faculty of Pharmacy, Al-Zaytoonah University of Jordan, Amman, Jordan; 3 Laboratory for Molecular Modeling, Division of Chemical Biology and Medicinal Chemistry, Eshelman School of Pharmacy, The University of North Carolina at Chapel Hill, Chapel Hill, North Carolina, United States of America; 4 Board Member, Jordan CDC, Amman, Jordan; Al-Azhar University / King Khalid University, EGYPT

## Abstract

Colorectal cancer (CRC) ranks as the third most prevalent cancer worldwide and the second leading cause of cancer-related deaths. Despite advances in treatment, drug resistance remains a critical challenge. Dipeptidyl peptidase 4 (DPP4), a multifunctional cell surface protein, shows variable expression across malignancies and plays a role in cancer biology. DPP4 inhibitors, initially developed as antidiabetic agents, have demonstrated anticancer properties in several cancer types. In this study, network pharmacology analysis revealed that DPP4 inhibitors modulate critical cancer-associated pathways, such as proteoglycans in cancer, ECM(extracellular matrix)-receptor interaction, and PI3K-AKT (phosphatidylinositol 3-kinase–protein kinase B) signaling. Experimental data showed dose-dependent growth inhibition in four CRC cell lines treated with FDA (Food and Drug Administration)-approved and novel DPP4 inhibitors. Combination treatments with doxorubicin yielded synergistic effects, whereas those with 5-fluorouracil (5FU) were either synergistic or additive. The examined DPP4 inhibitors effectively suppressed colony formation in HCT-116 cells and induced apoptosis. Additionally, the inhibitors resulted in G0/G1 cell cycle arrest and significantly reduced the expression of *CD26 (Cluster of Differentiation 26)*, *Bcl-2(B-cell lymphoma 2)*, and *VEGF (Vascular Endothelial Growth Factor)* in HCT-116 cells. Our findings highlight the potential therapeutic utility of DPP4 inhibitors in CRC treatment, either as standalone agents or in combination with standard chemotherapeutics. Moreover, the computational insights provided herein enhance our understanding of the molecular mechanisms underlying the anticancer effects of DPP4 inhibitors, paving the way for their potential clinical application.

## 1. Introduction

Cancer is a group of diseases characterized by uncontrolled cellular growth, local tissue invasion, and the spread of cancerous cells to other parts of the body [1]. Colorectal cancer (CRC) continues to be the second leading cause of cancer mortality worldwide [2]. In 2022, CRC was the third most diagnosed cancer in both males and females in the United States. It was associated with the third most significant percentage of cancer-related deaths among other cancers [3]. Adenocarcinoma is the most frequent type of CRC, however, Carcinoid tumors, sarcoma, and lymphoma are considered rarer subtypes [4].

Surgery, chemotherapy, radiotherapy, or a combination of these techniques are currently used to treat colorectal cancer [5]. Chemotherapy is the application of chemicals that have cytotoxic effects on rapidly growing cells [6]. Fluoropyrimidines were the backbone of colorectal cancer chemotherapy regimens during the past half-century. 5-fluorouracil (5FU), which is a thymidylate synthase inhibitor, has been the most widely used among fluoropyrimidines. [7]. Unfortunately, traditional chemotherapeutic agents often present with a package of impediments, such as significant side effects, multi-drug resistance (MDR), and high cost [8–10].

Over the last two decades, evidence has begun to accumulate supporting the role of glucose metabolism in cancer development and progression, and a solid relationship between diabetes and the occurrence of several types of cancers has been established [11,12]. Diabetes increases the risk of colon and rectum cancer by 1.2–1.5 times [13]. This association may be due to shared risk factors between the two diseases, including obesity, hyperinsulinemia, and insulin resistance [14].

Dipeptidyl peptidase-4 DPP4 is a plasma-soluble enzyme discovered in the gut mucosa’s capillary bed and other organs, such as the kidney and liver [15]. It is a type II transmembrane glycoprotein and related to the serine proteases family that stimulates the degradation of growth factors, chemokines, neuropeptides, and peptide hormones [16,17]. Interestingly, evidence is emerging to support the role of the DPP4 enzyme in the progression and metastasis of many cancer types, including CRC, hematological malignancies, thyroid cancer, and lung cancer, implying that DPP4 could be a promising therapeutic target for developing new cancer treatment strategies [18,19]. DPP4 has been proposed as a biomarker and prognostic factor in the survival of CRC patients and other diseases [20,21]. DPP4 inhibitors, also known as gliptins, are a group of oral antidiabetics licensed by the FDA for Type 2 Diabetes (T2D) treatment [22]. Several studies have recently been published that highlight the role of DPP4 inhibitors in tumorigenesis [23–25].

This study aims to investigate the potential of DPP4 inhibitors as therapeutic agents against colorectal cancer. The findings could contribute to the discovery of novel anticancer treatments and offer valuable insights into repurposing existing drugs as anticancer agents, potentially reducing production costs and accelerating drug development timelines.

## 2. Materials and methods

### 2.1. Computational methods

#### 2.1.1. *Network pharmacology approach.*

We applied an informatics workflow to study the network pharmacology of DPP4 inhibitors based on the methods developed by Hajjo et al. [26–28] to formulate testable hypotheses regarding the putative anticancer mechanisms of DPP4 inhibitors. This workflow incorporates two major components: (1) a network-building module to identify nearest neighbor proteins that have direct interactions with DPP4; and (2) a pathway enrichment module to elucidate the biological processes involved in the mechanism of action of the chemical compounds targeting DPP4.

#### 2.1.2. *Protein–protein interactions.*

A systematic search for DPP4 nearest neighbor (NN) genes/proteins was conducted in Cytoscape [29] version 3.10.3, using the STRING [30] protein query application with a medium confidence interaction score threshold of 0.400. The network was limited to the first shell of interactors with a maximum of 10 nodes to define the core high-confidence interactome of DPP4. All retrieved protein–protein interactions (PPIs), including both physical and functional interactions, were retrieved, and then the network-building tools in Cytoscape were utilized to generate DPP4’s PPI networks.

#### 2.1.3. *Enrichment analysis.*

Pathway enrichment analyses for the network nodes were performed using the STRING functional enrichment tool within Cytoscape [29] (version 3.10.3). Analyses were run against the KEGG Pathways, Gene Ontology Biological Processes, and Reactome databases. The significance of each enrichment result was assessed by calculating hypergeometric p-values, which were subsequently adjusted for multiple testing using the Benjamini-Hochberg method to generate False Discovery Rates (FDRs). Pathways and terms with FDR values below a threshold of 0.05 were considered statistically significant and selected for further biological investigation.

### 2.2. Experimental methods

#### 2.2.1. *Cell culture.*

Colorectal cancer cell lines: HCT116 (ATTC No. CCL-247), SW480 (ATTC No. CCL-228), SW620 (ATTC No. CCL-227) and Caco2 (ATTC No. HTB-37) cells were purchased from American Type Culture Collection (ATCC, USA)), and were grown in Dulbecco’s Modified Eagle’s Medium (DMEM) (Eurobio, French) supplemented with 10% Fetal bovine serum (FBS) (Capricorn Scientific GmbH, Germany), 100 U/mL penicillin (Euro Clone, Italy), 0.1 mg/mL streptomycin (Euro Clone, Italy), and 2 mM L-glutamine (Euro Clone, Italy) and were incubated in a humidified environment with 5% CO_2_ and 95% air, as previously described [31,32].

#### 2.2.2. *Compounds.*

The synthesized DPP4 inhibitors, N^4^-sulfonamido-succinamic, phthalamic, acrylic and benzoyl acetic acid derivatives, sulfamoyl-phenyl acid esters, were reported previously [33,34] and are shown in Table 1S in S1 File. All compounds were dissolved in DMSO to obtain a concentration of 30 mM, the final concentration of DMSO was ensured not to exceed 1%. Further dilution of stock solutions was carried out using DMEM medium to target concentrations immediately before use. Doxorubicin C_27_H_29_NO_11_·HCl [molar mass (MM), 580.0 g/mol] was purchased from Sigma-Aldrich (St. Louis, MO, USA; Cat. No. D1515). A stock solution was prepared (10 mM) and was diluted with DMEM immediately before use. 5-Fluorouracil (5-FU) was purchased from Sigma-Aldrich (St. Louis, MO, USA; Cat. No. F6627). A stock solution was prepared at a concentration of 100 mM, aliquoted, and stored at –20°C until use.

#### 2.2.3. *Cell viability assay.*

As previously described, [35], the 3-(4,5-dimethylthiazol-2-yl)-2,5-diphenyltetrazoliumbromide (MTT) test was used to assess cellular growth. CRC cells were seeded in 96-well plates at a density of 6000 cells for HCT116, 9000 cells for Caco2, and 7000 cells for SW620 and SW480. After 24 h, cells were treated with different concentrations of DPP4 inhibitors, doxorubicin, and 5-fluorouracil alone or in combination. After 48 h 10 µL of MTT dye at a working concentration of 5 mg/mL was added to each well, resulting in a final concentration of 0.5 mg/mL, followed by plate incubation for 3 h in the dark at 37°C in a humidified incubator set at 95% humidity and 5% CO_2_. After the incubation period had passed, the media was carefully aspirated, and formazan crystals were solubilized in 100 µL of DMSO; the plates were kept on an orbital shaker at 150 RPM for 15 minutes.

Optical density (OD) was measured at 570 nm using a microplate reader (µ Quant Plate Reader, Biotek, USA). Cell viability was determined and IC_50_, defined as the concentration of a compound required to inhibit cancer cell growth or viability by 50% under defined experimental conditions, was calculated using GraphPad Prism 7 software, (GraphPad Software, San Diego, USA). All experiments were run in duplicate wells and repeated twice.

#### 2.2.4. *Colony formation in soft agar assay.*

To perform the soft agar assay, A base layer of 0.5% (w/v) agar was prepared by adding autoclaved 1% (w/v) agar solution to 2x DMEM medium in a 1:1 ratio and allowed to solidify at room temperature [31,32]. A mixture of 1 × 10 ^^4^ of HCT116 cells previously treated with either IC_50_ or sub-IC_50_ (0.5 IC_50_ [36]) concentrations of drugs saxagliptin, sitagliptin, AE-AMID, and PA-AMID and 0.6% (w/v) agar solution was prepared in a 1:1 ratio and added to the top of the base layer, allowed to solidify. Each soft agar layer required 2 mL (1 mL media + 1 mL agar) to properly cover the 6-well surface. The plate was incubated for 2 weeks. Photos were captured after 12 days using the EVOS XL Core imaging system (Invitrogen, USA).

#### 2.2.5. *Flow cytometry analysis of apoptosis.*

HCT116 cells were plated in 6-well plates (2 replicate/group) in 3 mL medium and allowed to attach overnight. The cells were then treated with double IC_50_ concentration [37] of saxagliptin, sitagliptin, AE-AMID, and PA-AMID while doxorubicin was used as a positive control. Negative control wells contained fresh complete medium with DMSO at the same final concentration used in compound-treated wells. After 48 h of treatment, in a 5 mL flow tube, both floating and adhering cells (harvested using 500 µL of trypsin) were collected and centrifuged for 10 minutes at 1400 RPM, 4ºC, according to manufacturer protocol. The supernatant was discarded, and the cell pellet was re-suspended in 500 µL cold PBS and centrifuged to remove any remaining medium. Then, the pellets were resuspended again in 200 µL of 1X binding buffer per tube. The cells were then stained with 5 µL Annexin V-FITC and incubated at room temperature for 5 minutes, followed by the addition of 10 µL of propidium iodide (PI) (50 µg/mL) to each tube. The samples were analyzed immediately using BD ACSCanto II flow cytometer (BD Biosciences, USA) and the analysis of the result was performed using BD FACSDiva software.

#### 2.2.6. *Cell cycle analysis assay.*

HCT116 cells were plated in Petri dishes (2 replicate/group) in 5 mL medium and allowed to attach overnight in the humidified controlled temperature incubator set at 37°C, 95% humidity, and 5% CO_2_.Then cells were treated with sub-IC_50_ (0.5 IC_50_) concentration of saxagliptin, sitagliptin, AE-AMID, and PA-AMID for 48 h. Negative control wells contained fresh complete medium with DMSO at the same final concentration used in compound-treated wells. After the treatment time had passed, the cells were harvested by discarding the media and washing with 5 mL PBS followed by trypsinization. For this assay, a target of 1 × 10^6^ cell pellets in 500 μL PBS are indicated. The cells were then collected and fixed overnight at −20°C with 600 μL of 70% ice-cold ethanol.

Then the fixed cells were thawed and then washed with 1 mL of ice-cold 1X PBS twice each time interrupted by centrifugation, with the supernatant discarded. Then, samples were left to hydrate in 1mL of 1X, PBS at room temperature for 15 minutes. During the hydration time, a working concentration of 50 μg/mL of PI was prepared by diluting PI (1 mg/mL) in staining buffer by a 1:20 ratio, in addition to DNase-free RNase A (Sigma-Aldrich) to a final concentration of 5 μg/mL. Lastly, 300 μL of PI working solution was added to each tube and incubated in the dark at room temperature for 30 minutes. The samples were then analyzed using BD FACSCanto II flow cytometer (BD Biosciences, USA), and the analysis of the result was performed using BD FACSDiva software.

#### 2.2.7. *Quantitative Polymerase Chain Reaction (qPCR).*

HCT116, Caco2, SW480, and SW620 cells were seeded at a density of 3 × 10^5^ cells per well in a 6-well plate and incubated for 24 h. Then, HCT116 cells were treated with sub-IC_50_ (0.1 IC_50_ [32]) of saxagliptin, sitagliptin, AE-AMID, and PA-AMID for 48 h.

After the treatment period had elapsed, total RNAs of cells were purified using the Direct-zol™ RNA Miniprep Plus kit. After purification, the reverse-transcription reaction solution was prepared on ice by adding the components of ProtoScript First Strand cDNA Synthesis Kit with 500 ng of total RNAs, and RNase Free dH2O up to 20 μL. qPCR was performed using an SYBR Green Real-time PCR Master Mix and the Applied Biosystems 7500 real-time PCR detection system. The primer sequences used are shown in Table 2S in S1 File.

Thermal cycling conditions for CD26 were for amplification, cDNA was initially denatured at 95°C for 15 minutes, 50 cycles of 95°C for 10 sec, 59ºC for 30 sec, and 72°C for 30 sec. For B-cell lymphoma 2 (Bcl2), initial denaturation at 95°C for 15 minutes, followed by 45 cycles of 95°C for 10 sec, 59°C for 30 sec, and 72°C for 30 sec. For glyceraldehyde 3-phosphate dehydrogenase (GAPDH), initial denaturation at 95°C for 15 minutes, followed by 45 cycles for 95°C for 15 sec, 58°C for 30 sec, and 72°C for 30 sec. For vascular endothelial growth factor (VEGF), cDNA was initially denatured at 95°C for 15 minutes, followed by 40 cycles of 95°C for 10 seconds, 58°C for 30 seconds, and 72°C for 30 seconds.

#### 2.2.8. *Statistical analysis.*

Data analysis was performed using GraphPad Prism software (GraphPad Prism version 9.0.0 for Windows; GraphPad Software, San Diego, California, USA). An independent sample t-test, one-way ANOVA, or two-way ANOVA determined the differences between treatment groups. All experiments were run in duplicates and with three independent experiments. Data are expressed as mean ± SD, and *p* < 0.05 was considered a statistically significant difference. IC_50_ values were calculated using non-linear regression analysis. The combination index (CI) was calculated using CompuSyn software (Combosyn Inc., Paramus, NJ, USA), which is based on Chou-Talalay’s Combination Index Theorem, as shown in Equation 1.

Equation 1. Chou-Talalay’s Combination Index Theorem.

Where (Cx)1 = dose of compound 1 to produce 50% cell kill alone, (C)1 = dose of compound 1 to produce 50% cell kill in combination with (C)2. (Cx)2 = dose of compound 2 to produce 50% cell kill alone, (C)2 = dose of compound 2 to produce 50% cell kill in combination with (C)1.


$$CI\;=\;\frac{\left(C\right)1}{\left(Cx\right)1}+\frac{\left(C\right)2}{\left(Cx\right)2}\;$$

The fold reduction in IC₅₀ was determined by comparing the IC_50_ of the chemotherapy when used alone versus in combination with the examined compound. A higher fold reduction reflects enhanced potency in the combination. The percent reduction in IC₅₀ of the chemotherapy was calculated to express how much the IC₅₀ value decreased when the drug was used in combination, relative to its single use. Colony size, colony numbers, and band quantification were measured using ImageJ software version 1.53e.

## 3. Results

### 3.1. Network pharmacology of DPP4 inhibitors

**Fig 1:**
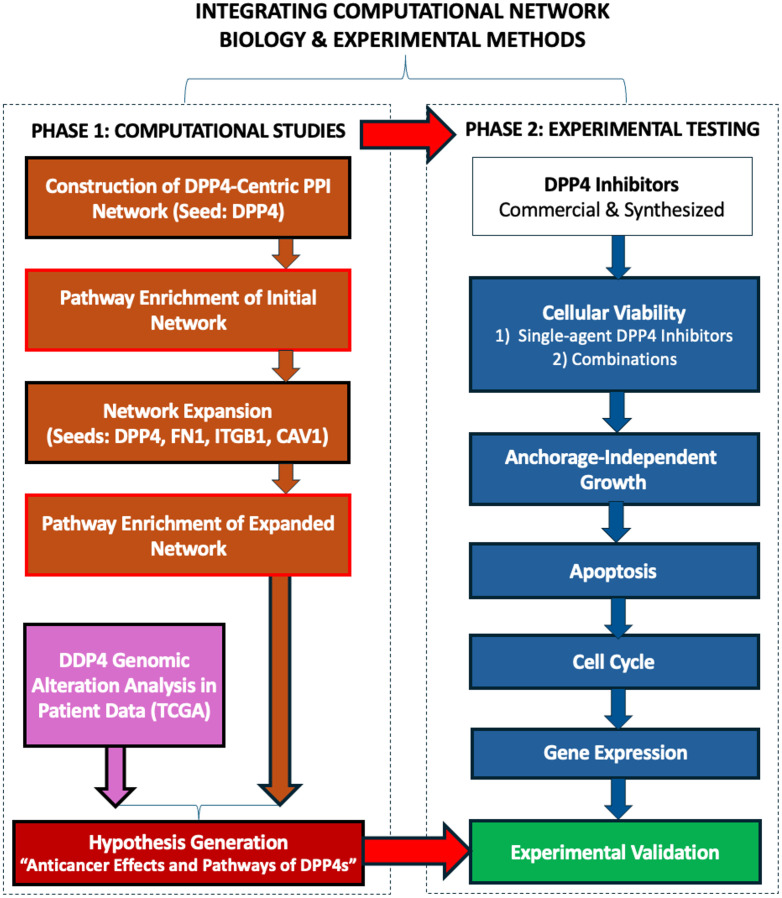
Workflow to integrate computational network biology and experimental testing for characterizing the anticancer activity of DPP4 inhibitors. The schematic outlines the two-phase strategy of this study. Phase 1 (Computational Studies) begins with the construction of a DPP4-centric protein-protein interaction (PPI) network using Cytoscape and the STRING database (confidence score of selected nodes ≥ 0.4). Pathway enrichment analysis of this network identifies putative cancer-associated pathways. Key hub genes from this initial analysis are selected to generate an expanded PPI network, which undergoes a second enrichment analysis to pinpoint central mechanisms. Concurrently, the genomic alteration landscape of DPP4 is analyzed in patient data from TCGA. These analyses collectively generate a testable hypothesis on the anticancer pathways of DPP4 inhibitors. Phase 2 (Experimental Testing) involves the validation of these computational predictions. A suite of in vitro assays—including cellular viability, anchorage-independent growth, apoptosis, cell cycle analysis, and gene expression profiling—is performed using both commercial and synthesized DPP4 inhibitors to functionally validate the hypothesized mechanisms.

**Fig 2:**
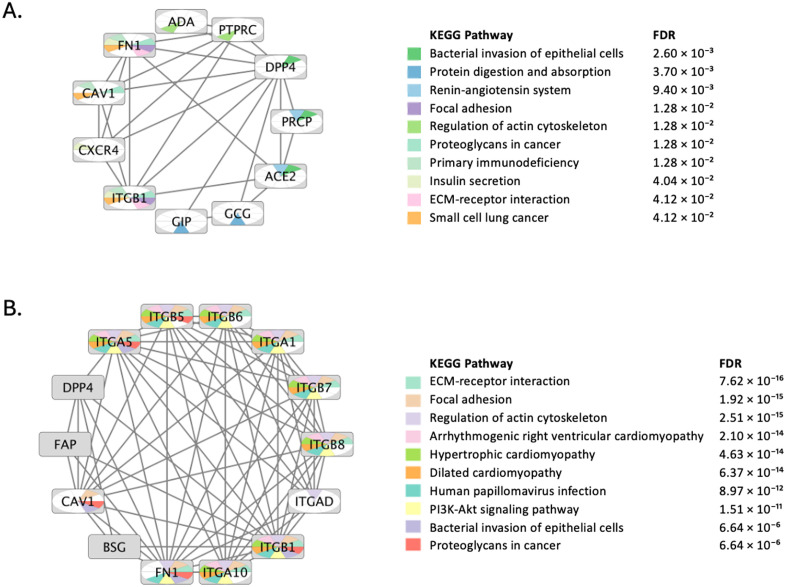
Protein-protein interaction networks for DPP4. A) Initial direct interactions network using DPP4 as a seed querying the STRING database with a medium confidence score threshold of 0.400 and limited to 10 first-shell interactors to define a high-confidence core interactome. The top 10 significantly enriched KEGG pathways (FDR ≤ 0.05) are displayed. The FDR value for each pathway is indicated. Pathways strongly associated with cancer mechanisms, such as ‘Proteoglycans in cancer’, ‘Focal adhesion’, and ‘ECM-receptor interaction’, are prominently featured. B) Expanded direct interactions network using DPP4 plus three additional gene seeds identified from the initial network: FN1, ITGB1 and CAV1. The additional seeds were selected based on their high connectivity (hub status) in the first network and their established biological relevance to the enriched cancer pathways. The same confidence and interactor limits were applied. Enrichment analysis of the expanded network confirmed the cancer-related pathways with greater statistical robustness and identified the PI3K-Akt signaling pathway as the most significantly enriched.

A network pharmacology approach was employed to hypothesize the anticancer effects and associated pathways of DPP4 inhibitors, as shown in Fig 1. Initially, a protein-protein interaction (PPI) network for DPP4 was constructed using Cytoscape version 3.10.3 and the STRING protein query tool. The network was generated by querying DPP4 as a single node, with an intermediate selectivity threshold of 0.5 to capture a broader set of hub proteins. This approach aimed to identify potential cancer-related pathways and form hypotheses regarding the anticancer potential of DPP4 inhibitors. A maximum of 10 proteins were retrieved for further analysis. The resulting network (Fig 2A) was then subjected to enrichment analysis against a comprehensive collection of pathway databases that are part of Cytoscape’s STRING App [7].

Enrichment analysis revealed several significant cancer-related pathways, including ‘proteoglycans in cancer’ and ‘small cell lung cancer pathway’. Other enriched pathways were strongly associated with cancer, such as the extracellular matrix (ECM) receptor interaction pathway, which plays a pivotal role in tumorigenesis, metastasis, and drug resistance. Additionally, pathways like focal adhesion and actin cytoskeleton regulation were identified as crucial for tumor progression, metastasis, and angiogenesis. Disruptions in integrin signaling, focal adhesion kinase (FAK), and Rho GTPases, coupled with aberrant actin dynamics, contribute to enhanced cancer cell motility, invasion, and therapeutic resistance.

Subsequently, a second interaction network was generated using DPP4 and three additional seed genes/proteins: caveolin 1 (CAV1), fibronectin 1 (FN1), and integrin subunit beta 1 (ITGB1). These genes were selected from the first network as they represented top hub nodes with high degree centrality and are known to be critically involved in cancer-relevant pathways (as revealed in the initial network). The new network (Fig 2B) was constructed with a selectivity threshold of 0.5 and a maximum of 10 nodes. Enrichment analysis of this network highlighted the PI3K-AKT signaling pathway, which displayed a highly significant FDR value of 1.51 × 10 ⁻ ¹¹. This suggests that the PI3K-AKT pathway may play a key role in the anticancer mechanisms of DPP4 inhibitors. Although other pathways are also likely involved, network analysis ranks PI3K-AKT as the most significant. Pathways previously enriched in Fig 2A, including ECM-receptor interaction, focal adhesion, and actin cytoskeleton regulation, were again enriched in Fig 2B, but with more robust FDR values, further supporting their relevance to cancer progression.

**Fig 3:**
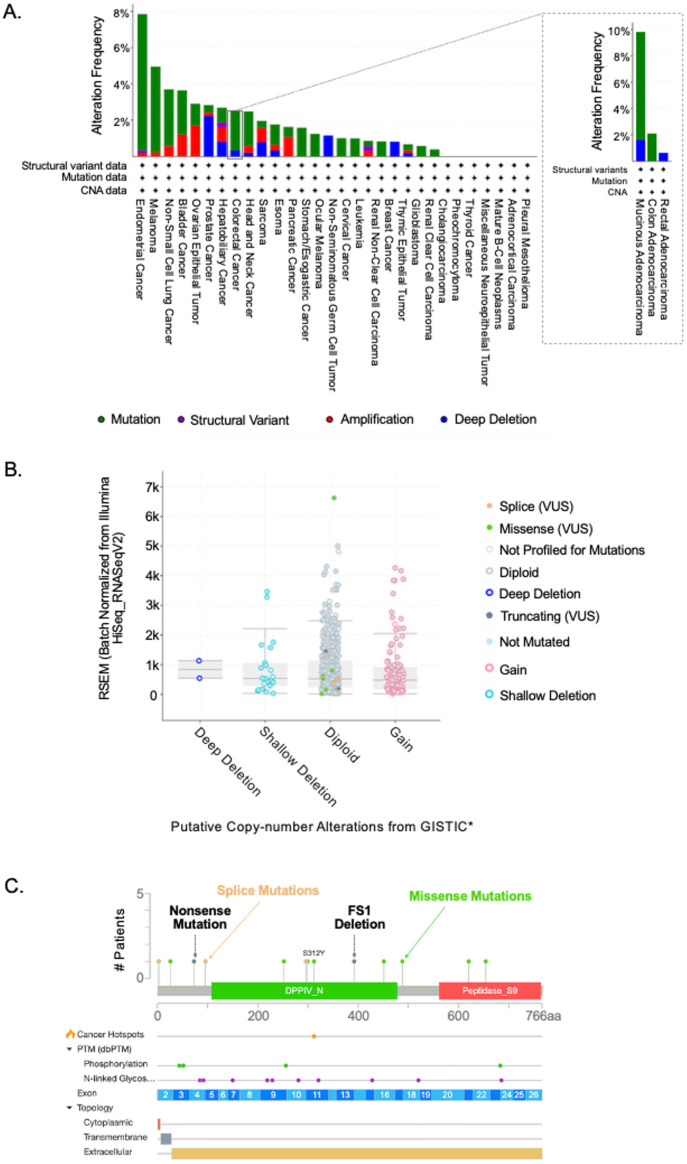
Genomic alterations of DPP4 in cancer. A) Genetic alterations of DPP4 across various cancer types, derived from a dataset of 10,967 samples from 10,953 cancer patients. B) mRNA expression levels of DPP4 in colorectal cancer patients, based on 594 samples. C) Frequency of DPP4 genetic mutations in colorectal cancer patients, analyzed from 594 samples. All patient data were sourced from TCGA PanCancer Atlas Studies [38] and analyzed using cBioPortal [39]. Source: National Cancer Institute as the source.

Targeting cancer pathways with DPP4 inhibitors holds significant promise as a therapeutic strategy to inhibit tumor growth and metastasis, and overcome therapeutic resistance, making it a compelling focus in the field of cancer research and drug development. DPP4, as a crucial regulator of several signaling pathways, may play a pivotal role in modulating cancer cell proliferation, migration, and response to treatment. However, it is essential to recognize that cancer patient data obtained from The Cancer Genome Atlas (TCGA) revealed that DPP4 genetic alterations were present in only 15 out of 594 colorectal cancer (CRC) patient samples (approximately 3%). Fig 3 summarizes the genomic alterations analysis of DPP4 in Cancer using the TCGA [35,36] database and cBioportal [36]. Our findings underscore the importance of personalizing cancer treatments with DPP4 inhibitors, considering the genetic status of DPP4 alterations within individual patients. Specifically, the efficacy of DPP4-targeted therapies may be more pronounced in patients with DPP4 mutations or alterations, while those without such alterations may be refractory to such treatments. As such, further studies to assess DPP4 expression, mutation status, and pathway involvement in various cancers are needed to refine the use of DPP4 inhibitors in clinical settings, ensuring their application is both targeted and optimized for specific patient populations.

In parallel, careful consideration of cancer cell line models in preclinical studies is crucial to ensure that the anticancer effects of DPP4 inhibitors are assessed in the most relevant biological context. Selecting appropriate cancer cell lines that accurately reflect the genetic alterations and pathway dependencies of the target cancer will be key to optimizing the translational potential of DPP4-targeted therapies.

### 3.2. Effect of DPP4 inhibitor treatment on the viability of colorectal cancer cell lines

To assess the impact of the synthesized DPP4 inhibitors on the viability of various colorectal cancer (CRC) cell lines, an MTT assay was performed on SW620, HCT116, SW480, and Caco2 cells treated with increasing concentrations (1–200 µM) of the inhibitors for 48 hours. The compounds exhibited antiproliferative effects, as evidenced by a reduction in cell viability in treated cells compared to untreated controls. The IC_50_ values, representing the concentration required to inhibit cell viability by 50%, are presented in Table 1.

**Table 1:** The 50% inhibitory concentration (IC_50_, µM) values for DPP4 inhibitor treatment in SW620, HCT116, SW480, and Caco2 colorectal cancer cells for 48 hours. Values are expressed as the mean ± SD of three independent experiments in duplicates (n = 6).

Compound	SW620	HCT-116	SW480	Caco-2
**PA-AMID**	158.2 ± 2.647	166.4 ± 0.849	239.3 ± 3.617	232.3 ± 1.96
**SA-PYR**	116.4 ± 3.748	179 ± 4.273	196.7 ± 2.505	308.1 ± 3.041
**SA-H**	86.01 ± 3.896	176 ± 6.99	237.2 ± 1.96	256.1 ± 2.087
**BA-PYR**	166.4 ± 5.192	193.2 ± 2.475	414.2 ± 3.253	>1000
**BA-THIAZ**	128.4 ± 1.95	132.3 ± 5.485	298 ± 5.515	228.4 ± 6.697
**AA-THIAZ**	83.77 ± 2.559	551.4 ± 6.435	339.3 ± 6.718	153.8 ± 4.97
**PE-H**	136.9 ± 0.566	378.3 ± 2.546	332 ± 6.576	435.5 ± 1.041
**AE-AMID**	189.2 ± 5.303	210.6 ± 2.081	246 ± 3.96	256.8 ± 1.889
**Saxagliptin**	168.6 ± 2.849	500.7 ± 1.526	288.4 ± 2.788	236.7 ± 4.101
**Sitagliptin**	103.2 ± 1.485	309.4 ± 1.677	238 ± 3.728	203.1 ± 2.697

### 3.3. Effect of combined treatment of DPP4 inhibitors and chemotherapy on the viability of colorectal cancer cell lines

**Fig 4:**
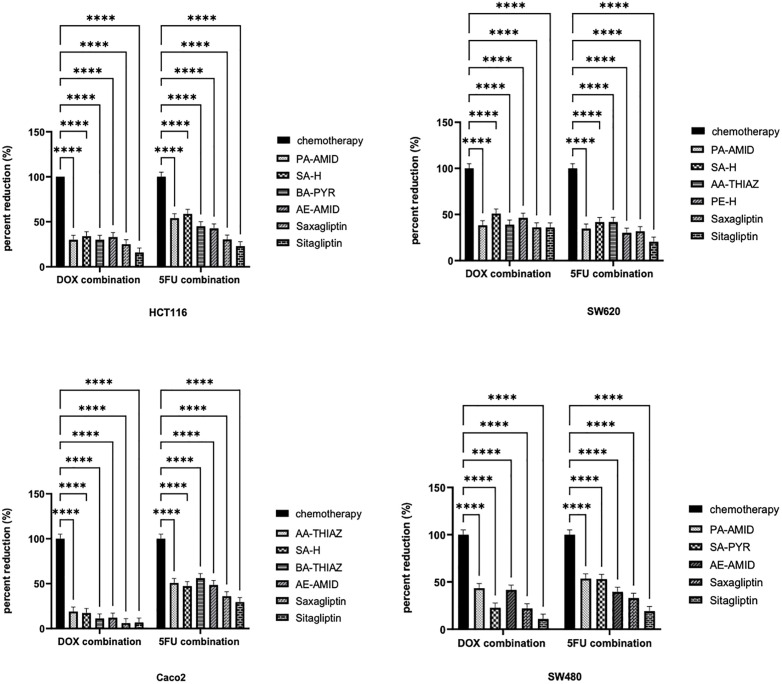
The percentage reduction of IC_50_ for chemotherapy in combination with DPP4 inhibitor treatment in HCT116, SW620, Caco2, and SW480 colorectal cancer cells. Cells were cultured and allowed to attach overnight. The next day, cells were treated with different concentrations of selected compounds of DPP4 inhibitors with either doxorubicin or 5-FU, for 48 h. After that, the cell viability was determined using an MTT assay. *P < 0.05 significantly different from respective cisplatin treatment. ****P < 0.0001.

To evaluate the impact of combining DPP4 inhibitors with chemotherapeutic agents on colorectal cancer (CRC) cells, each cell line was treated with selected DPP4 inhibitors in combination with either doxorubicin or 5-FU at a 1:1 IC_50_ ratio for 48 hours. The resulting combination index (CI) values are presented in Tables 2 and 3. Overall, the combined treatments demonstrated synergistic or additive effects. Fig 4 illustrates the percentage reduction in the IC_50_ values of the chemotherapeutic agents when used in combination with DPP4 inhibitors under the same treatment conditions.

**Table 2:** Combination index (CI) values for combined treatment of DPP4 inhibitors and doxorubicin in CRC cell lines. CI < 1, = 1, and > 1 indicate synergistic, additive, and antagonistic effects, respectively. µM: micromolar.

HCT 116			
Compound	IC_50_ (µM)	CI	Fold reduction
**Doxorubicin**	0.215		
**Doxorubicin: PA-AMID**	0.064	0.602	3.361
**Doxorubicin: SA-H**	0.073	0.702	2.937
**Doxorubicin: AE-AMID**	0.071	0.694	3.019
**Doxorubicin: Saxagliptin**	0.055	0.510	3.918
**Doxorubicin: Sitagliptin**	0.035	0.329	6.226
**Doxorubicin: BA-PYR**	0.065	0.550	3.331
**SW620**			
**Doxorubicin**	0.219		
**Doxorubicin: PA-AMID**	0.083	0.758	2.618
**Doxorubicin: SA-H**	0.113	1.028	1.933
**Doxorubicin:AA-THIAZ**	0.087	0.783	2.519
**Doxorubicin: Saxagliptin**	0.079	0.707	2.766
**Doxorubicin: Sitagliptin**	0.078	0.687	2.785
**Doxorubicin: PE-H**	0.102	0.924	2.151
**Caco2**			
**Doxorubicin**	23.999		
**Doxorubicin: SA-H**	4.133	0.340	5.806
**Doxorubicin:AE-AMID**	2.901	0.230	8.272
**Doxorubicin:AA-THIAZ**	4.537	0.374	5.289
**Doxorubicin: Saxagliptin**	1.444	0.114	16.623
**Doxorubicin: Sitagliptin**	1.597	0.132	15.025
**Doxorubicin:BA-THIAZ**	2.696	0.219	8.902
**SW480**			
**Doxorubicin**	0.648		
**Doxorubicin: PA-AMID**	0.282	0.872	2.299
**Doxorubicin: AE-AMID**	0.269	0.829	2.406
**Doxorubicin: Saxagliptin**	0.148	0.4582	4.375
**Doxorubicin: Sitagliptin**	0.071	0.230	9.182
**Doxorubicin: SA-PYR**	0.147	0.457	4.405

**Table 3:** Combination index (CI) values for combined treatment of DPP4 inhibitors and 5FU in CRC cell lines. CI < 1, = 1, and > 1 indicate synergistic, additive, and antagonistic effects, respectively. µM: micromolar.

HCT 116			
Compound	IC_50_ (µM)	CI	Fold reduction
**5FU**	22.198		
**5FU: PA-AMID**	11.984	1.110	1.852
**5FU: SA-H**	13.041	1.200	1.702
**5FU: AE-AMID**	9.477	0.909	2.342
**5FU: Saxagliptin**	6.740	0.603	3.293
**5FU: Sitagliptin**	5.324	0.483	4.169
**5FU: BA-PYR**	10.003	0.938	2.219
**SW620**			
**5FU**	21.854		
**5FU: PA-AMID**	7.599	0.680	2.876
**5FU: SA-H**	9.112	0.841	2.398
**5FU: AA-THIAZ**	9.150	0.848	2.388
**5FU: Saxagliptin**	6.931	0.632	3.153
**5FU: Sitagliptin**	4.495	0.439	4.862
**5FU: PE-H**	6.605	0.591	3.309
**Caco2**			
**5FU**	225.258		
**5FU: SA-H**	106.366	0.905	2.118
**5FU:AE-AMID**	109.278	0.895	2.061
**5FU: AA-THIAZ**	114.282	1.024	1.971
**5FU: Saxagliptin**	81.101	0.679	2.778
**5FU: Sitagliptin**	66.277	0.635	3.398
**5FU:BA-THIAZ**	126.327	1.116	1.783
**SW480**			
**5FU**	46.022		
**5FU: PA-AMID**	24.681	1.076	1.865
**5FU: AE-AMID**	18.195	0.785	2.529
**5FU: Saxagliptin**	15.197	0.662	3.028
**5FU: Sitagliptin**	8.809	0.405	5.224
**5FU: SA-PYR**	24.413	1.089	1.885

### 3.4. Effect of DPP4 inhibitor treatment on the colony formation ability of colorectal cancer

**Fig 5:**
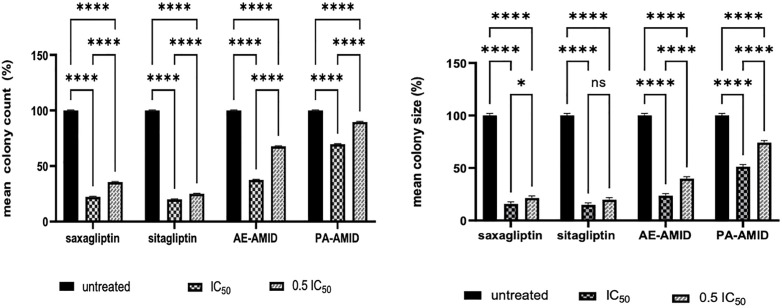
Effect of DPP4 inhibitors (Saxagliptin, Sitagliptin, AE-AMID, and PA-AMID) on colony count and size of HCT116 using colony formation assay. *P*-value < 0.05 expresses significantly different from respective untreated cells’ status; while asterisk: ns (not-significant) *P* > 0.05; * *P* ≤ 0.05; ** *P* ≤ 0.01; *** *P* ≤ 0.001; **** *P* ≤ 0.0001 (according to GraphPad Prism 9). The standard deviation of values did not exceed 5%. IC_50_: The 50% inhibitory concentration.

To investigate the effect of DPP4 inhibitor treatment on the colony-forming capability of CRC, HCT116 cells were treated with IC_50_ and sub-IC_50_ (0.5 IC_50_) concentrations of saxagliptin, sitagliptin, AE-AMID, and PA-AMID for 48 h. Afterward, cells were grown in soft agar. The results showed that DPP4 inhibitor treatment significantly *P* (≤ 0.0001) reduced the ability of HCT-116 cells to form colonies by diminishing the number and size of colonies compared to control cells. Representative images were taken on day 14 and are shown in Figure 1S in S1 File. The average colony size and the number of colonies formed in treated cells, compared to the control group, are presented in Fig 5.

### 3.5. Effect of DPP4 inhibitors on the cell cycle of colorectal cancer cell lines

**Fig 6:**
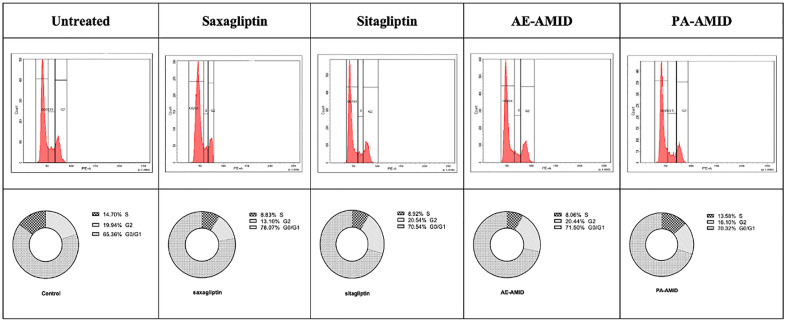
Histogram and partial summary of the effects of sub-IC₅₀ concentrations of Saxagliptin, Sitagliptin, AE-AMID, and PA-AMID on HCT116 cell cycle progression after 48 hours of treatment. DNA content was analyzed by flow cytometry following PI staining, with histograms indicating the distribution of cells across G0/G1, S, and G2/M phases. Gating was performed based on fluorescence intensity profiles, using the untreated control as a reference. Minor adjustments were made to accommodate treatment-induced shifts while maintaining consistency in phase identification.

To investigate the impact of DPP4 inhibitors on the cell cycle of colorectal cancer (CRC) cells, HCT116 cells were cultured in Petri dishes until near confluency, allowed to adhere overnight, and then treated with saxagliptin, sitagliptin, AE-AMID, or PA-AMID at sub-IC_50_ concentrations (0.5 × IC_50_) for 48 hours. Following treatment, cells were stained with propidium iodide as outlined in the methods section. All DPP4 inhibitors induced cell cycle arrest at the G0/G1 phase, as shown in Fig 6.

### 3.6. Effect of DPP4 inhibitors on apoptosis of colorectal cancer cell lines

**Fig 7:**
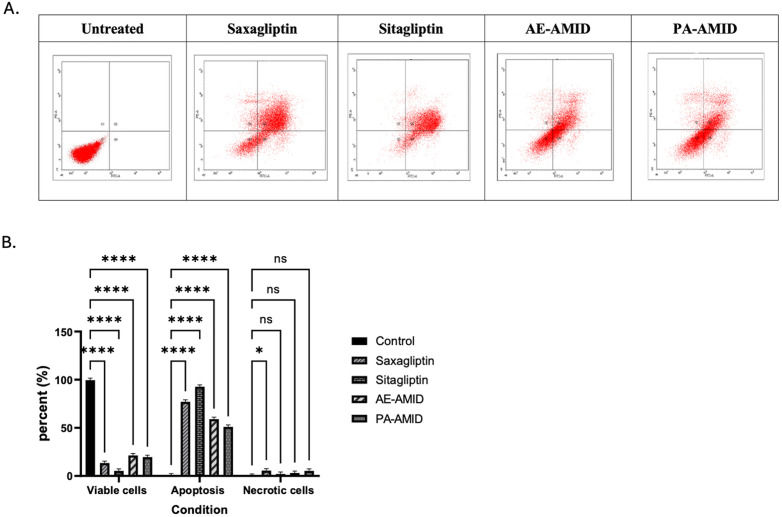
A) Dot plot for annexin V-FITC/ PI staining expressing the apoptotic effect of DPP4 inhibitors, Saxagliptin, Sitagliptin, AE-AMID, and PA-AMID (double IC_50_) for 48 hours treatment against HCT116 cells. Where Q3 showed viable cells, Q1 necrotic cells, Q2 late apoptotic, and Q4 early apoptotic. B) Percentages of healthy, apoptotic, and necrotic cells expressed as mean, SD did not exceed 5%. *P*-value <0.05 indicates statistical significance in comparison to untreated control, while asterisk: ns (not-significant) *P* > 0.05; * *P* ≤ 0.05; ** *P* ≤ 0.01; *** *P* ≤ 0.001; **** *P* ≤ 0.0001 (according to GraphPad Prism 9).

The Annexin V-FITC/Propidium Iodide apoptosis assay was employed to determine whether DPP4 inhibitors reduce the viability of HCT116 colorectal cancer cells through the induction of apoptosis or necrosis. Treatment with double the IC_50_ concentration of saxagliptin, sitagliptin, AE-AMID, and PA-AMID led to a significant increase (P < 0.0001) in both early and late apoptotic cell populations (Q2 + Q4 regions in the dot plot) compared to untreated controls, as illustrated in Fig 7A, B.

### 3.7. Effect of DPP4 inhibitors on the multiple gene expression of colorectal cancer cell lines

**Fig 8:**
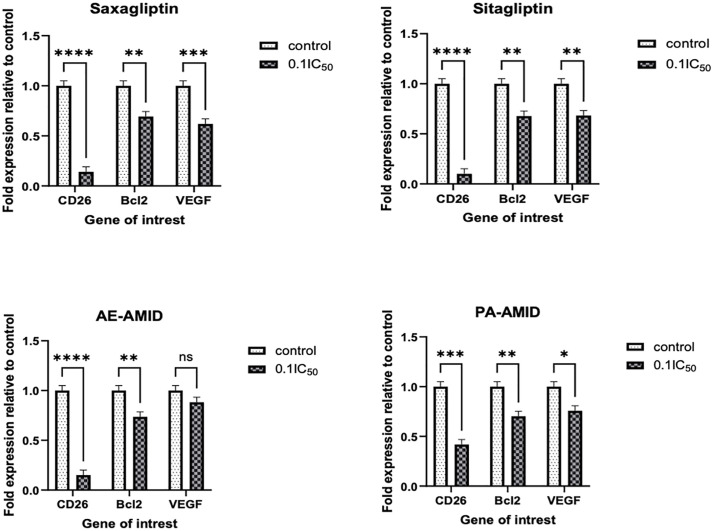
Effect of DPP4 inhibitors Saxagliptin, Sitagliptin, AE-AMID, and PA-AMID on multiple RNA expression of specific genes in HCT116 colorectal cancer cell line. *CD26*: the gene for dipeptidyl peptidase enzyme; *Bcl-2*: the gene for Bcl-2; *VEGF*: the gene for vascular endothelial growth factor; Fold difference expressed as mean±SD and was measured using ΔΔCt method. All experiments were run in duplicates and with three independent experiments. *P*-value < 0.05 express significantly different from respective untreated cells’ status; while asterisk: ns (not-significant) *P* > 0.05; * *P* ≤ 0.05; ** *P* ≤ 0.01; *** *P* ≤ 0.001; **** *P* ≤ 0.0001 (according to GraphPad prism 9). IC_50_: The 50% inhibitory concentration.

To assess the impact of DPP4 inhibitors on gene expression in HCT116 cells, the mRNA levels of CD26, Bcl-2, and VEGF were evaluated by qPCR following 48-hour treatment with saxagliptin, sitagliptin, AE-AMID, and PA-AMID at 0.1 × IC_50_ concentrations. Expression levels were compared to those in untreated control cells. Saxagliptin, sitagliptin, and PA-AMID significantly (P < 0.01) downregulated CD26, Bcl-2, and VEGF expression. AE-AMID significantly reduced CD26 and Bcl-2 expression but had no significant effect on VEGF, as presented in Fig 8.

## 4. Discussion

This study utilized a network pharmacology approach to investigate the anticancer potential of DPP4 inhibitors, identifying several key cancer-related pathways that may be modulated by DPP4. Protein-protein interaction (PPI) network analysis revealed critical pathways, including proteoglycans in cancer, ECM-receptor interaction, focal adhesion, and actin cytoskeleton regulation, all of which play pivotal roles in tumorigenesis, metastasis, and therapeutic resistance. These findings align with previous studies suggesting that DPP4 may contribute to cancer progression by regulating essential processes such as cell migration, proliferation, and invasion. Further analysis, incorporating additional seed genes (CAV1, FN1, and ITGB1), strengthened the relevance of these pathways and highlighted the PI3K-AKT signaling pathway as a central mechanism through which DPP4 inhibitors could exert their anticancer effects. This underscores the PI3K-AKT pathway as a critical target for DPP4 inhibitors in modulating cancer cell survival and growth. Building on these network biology and pathway analysis insights, the study further evaluated the anticancer effects of various DPP4 inhibitors in four colorectal cancer (CRC) cell lines, including HCT-116, SW480, SW620, and Caco-2.

Using both FDA-approved and novel DPP4 inhibitors, this study aimed to assess the role of the DPP4 enzyme in CRC progression through antiproliferative and anti-tumorigenic assays. Molecular changes induced by DPP4 inhibitors were also investigated, with a focus on the mechanisms of apoptosis and cell cycle alterations in CRC cells, particularly in the HCT-116 cell line. These findings provide valuable insights into the transcriptional mechanisms by which different DPP4 inhibitors might modulate cancer progression and cell death pathways, offering a deeper understanding of their potential therapeutic applications.

The colony formation assay, cell cycle assay, apoptosis assay, and qPCR were performed using the HCT-116 cell line due to its ease of handling, short doubling time, and robust colony-forming capability. Additionally, HCT-116 cells are known to exhibit high CD26 gene expression, making them a suitable model for studying DPP4-related effects in cancer [40]. The DPP4 inhibitors used in this study effectively inhibited the proliferation of all four colorectal cancer (CRC) cell lines (HCT-116, SW480, SW620, and Caco-2) in a concentration-dependent manner, compared to the untreated control cells. These antiproliferative effects may be linked to the inhibition of PI3K-AKT signaling, as suggested by the enrichment analysis of DPP4-associated networks, which highlighted this pathway as a key player in the anticancer mechanisms of DPP4 inhibitors. The observed results align with previous studies reporting the anti-proliferative activity of DPP4 inhibitors in various cancer types, including colorectal [25,41], gastric cancer cells [42] thyroid [43] breast [44] and endometrial cancer [45]. Notably, the IC_50_ values varied across drug agents and cell lines, emphasizing the importance of considering drug specificity and cell line variability in evaluating DPP4 inhibitors’ therapeutic potential.

The molecular changes induced by DPP4 inhibitors were also investigated, with a focus on the mechanisms of apoptosis and cell cycle alterations in CRC cells, particularly in the HCT-116 cell line. These findings provide valuable insights into the transcriptional mechanisms by which different DPP4 inhibitors might modulate cancer progression and cell death pathways, offering a deeper understanding of their potential therapeutic applications.

Furthermore, the potential for combination therapy with conventional chemotherapeutic agents, such as 5FU and doxorubicin, was explored. 5FU has traditionally been the principal chemotherapy medication used to treat metastatic CRC [46]. 5FU causes cytotoxicity by interfering with critical biosynthetic activities by blocking thymidylate synthase (TS) or misincorporating its metabolites into RNA and DNA [47]. Unfortunately, 5FU treatment has several drawbacks, including a short half-life, significant cytotoxicity, low bioavailability, and chemoresistance development [47,48]. The combination of DPP4 inhibitors with 5FU reduced the concentration of 5FU needed to produce an anti-proliferative effect in colorectal cancer cells (synergistic or additive effect, CI<=1), which may reduce the dose resistance and the dose-dependent toxicity. The results of Zheng et.al revealed that DPP4 was highly elevated in HCT-8/5-FU treated cells, and was linked to 5FU resistance in colon cancer cells, and the inhibition of DPP4 using sitagliptin or vildagliptin could attenuate the angiogenesis of the resistant cells [49].

Doxorubicin is a globally effective chemotherapeutic drug used to treat various cancers, including lung, multiple myeloma, and thyroid tumors. Doxorubicin causes cytotoxicity by inhibiting topoisomerase II or generating free radicals [50]. Interestingly, combining DPP4 inhibitors with doxorubicin reduced the concentration of doxorubicin needed to produce an anti-proliferative effect in CRC cancer cells (synergistic effect, CI < 1). There is a well-known anti-inflammatory action of DPP4 inhibitors on nephropathies or cardiopathies associated with doxorubicin use [51,52]. To our knowledge, no present evidence has been established for combining DPP4 inhibitors with doxorubicin in cancer cells. In our combination assay, the selected DPP4 inhibitor compounds have the lowest IC_50_ values on each cell line from different chemical scaffolds. Based on our results, the combination of 5FU or doxorubicin with DPP4 inhibitors may contribute to reducing drug resistance and side effects associated with both chemotherapies. This synergy may be, in part, explained by the ability of DPP4 inhibitors to modulate the PI3K-AKT pathway, which is known to contribute to chemoresistance in CRC cells. However, it is important to note that while the combination of DPP4 inhibitors with chemotherapy is generally synergistic, the combination index can vary depending on the specific drug and cancer cell line used. This variability suggests that the efficacy of DPP4 inhibitors in combination therapy is influenced by factors such as the cellular context, drug-specific mechanisms, and the underlying pathways driving resistance in different cancer types. As such, the therapeutic effects of DPP4 inhibitors may differ across cancer cell lines and should be carefully considered when designing combination therapies.

In CRC cancer, the macrophage colony-stimulating factor (MCSF) is shown to be overexpressed. Overexpression of MCSF is frequently linked to lymph node metastasis and a poor prognosis in cancer patients [53]. The DPP4 inhibitors saxagliptin, sitagliptin PA-AMID, and AE-AMID significantly inhibited the ability of HCT116 cells to form colonies. These findings are compatible with the previously reported finding that sitagliptin inhibited the colony formation of breast and gastric cancer cells [54,42].

In CRC, the high expression of CD26 is associated with advanced stages of the disease, distant metastasis, and a poor prognosis [55,21]. The level of expression differs from one cell line to another. HT29 and HCT116 have higher expression levels compared to SW620 and SW480, which is compatible with Varela-Calviño *et al.‘s* findings [40]. DPP4 inhibitors PA-AMID, AE-AMID, saxagliptin, and sitagliptin resulted in a significant (P ≤ 0.001) reduction in the expression of CD26. The results of Pinheiro et.al on human peripheral blood mononuclear cells (PBMC) showed that sitagliptin treatment inhibited the proliferation of PBMC-stimulated cells in a dose-dependent manner and decreased CD26 gene expression by these cells, suggesting that sitagliptin may interfere with CD26 expression, dimerization, and cell signaling [56].

Angiogenesis is important in the progression of colorectal cancer. VEGF appears to be the most important angiogenic factor in human colorectal cancer and is linked to metastasis and a poor prognosis [57]. In this study, PA-AMID, saxagliptin, and sitagliptin significantly reduce the expression of VEGF. Previously sitagliptin showed a significant reduction in hepatic content of VEGF in mice with Hepatocellular carcinoma (HCC) [58].

The common mechanism of anti-proliferative agents is the induction of cell cycle arrest at a specific checkpoint and initiation of apoptosis (programmed cell death) [59]. Sitagliptin, saxagliptin, and AE-AMID resulted in a significant (p-value < 0.0001) increase in early and late apoptosis in CRC-exposed compared to untreated control groups. Bcl-2 inhibits apoptosis [60], sitagliptin, saxagliptin, and AE-AMID resulted in a significant (p-value < 0.01) decrease in Bcl-2 gene expression, which is consistent with the apoptotic effect of these DPP4 inhibitors. Sarker *et al*. found that sitagliptin has a potent cytotoxic effect on both types of cancer cells (MCF7 and HepG2). It has also shown a certain impact on both early and late apoptogenic efficacy in HepG2 and late apoptogenic efficacy in MCF7 [61].

After DNA damage, a ‘checkpoint’ controls the cell cycle’s arrest, allowing repair to occur before mutations can be generated, or causing apoptosis to protect the normal functions in the cells [62]. Apoptosis is controlled by genes involved in cell cycle progression; therefore, they are interconnected [63]. In our study, the cell cycle assay results suggest that treatment of all DPP4 inhibitors with a sub-IC_50_ concentration on HCT116 resulted in G0/G1 phase arrest.

## 5. Conclusion

This study highlights the promising therapeutic potential of DPP4 inhibitors in colorectal cancer (CRC) treatment. Through network pharmacology analysis, we identified their ability to modulate critical cancer-associated pathways, including proteoglycans in cancer, ECM-receptor interaction, and PI3K-AKT signaling. Experimental evidence demonstrated their efficacy in inhibiting CRC cell growth, inducing apoptosis, and arresting the cell cycle, as well as reducing key oncogenic markers such as CD26, Bcl-2, and VEGF. Notably, synergistic effects were observed when DPP4 inhibitors were combined with doxorubicin, while combinations with 5-fluorouracil showed either synergistic or additive effects.

These findings underscore the potential of DPP4 inhibitors as viable therapeutic agents for CRC, either as standalone treatments or in combination with existing chemotherapeutics. Additionally, the mechanistic insights gained through computational and experimental approaches provide a strong foundation for further preclinical and clinical studies, paving the way for their integration into CRC treatment strategies.

## Supporting information

S1 File Contains Table 1S (chemical structures of DPP4 inhibitors), Table 2S (primers’ forward and reverse sequences with their optimized annealing temperature), and Figure 1S (representative cell colonies in soft agar for HCT116 cells treated with Saxagliptin, Sitagliptin, AE-AMID, and PA-AMID). (DOCX)
