# Supplementary material for: DPP4 inhibitors as a novel therapeutic strategy in colorectal cancer: Integrating network biology and experimental insights
Source: PLoS One. 2025 Oct 10;20(10):e0334223. doi: 10.1371/journal.pone.0334223 (PMC12513607; doi:10.1371/journal.pone.0334223)
Supplement: S1 File — (DOCX) [file pone.0334223.s001.docx]

**Table 1S. Chemical structures of DPP4 inhibitors.**

| **Compound** | **Molecular weight** | **Chemical structure** | **R group** |
| --- | --- | --- | --- |
| PA-AMID | 362.36 | 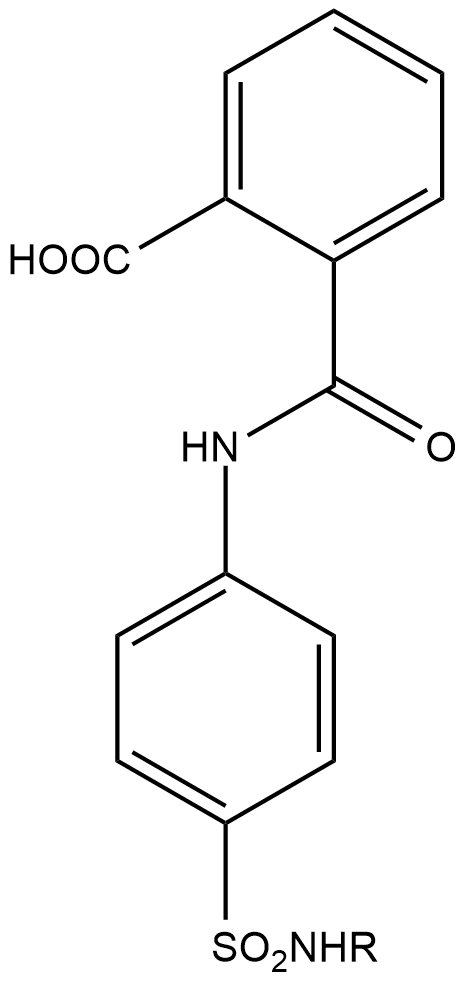 | R=amidino |
| SA-PYR | 350.35 | 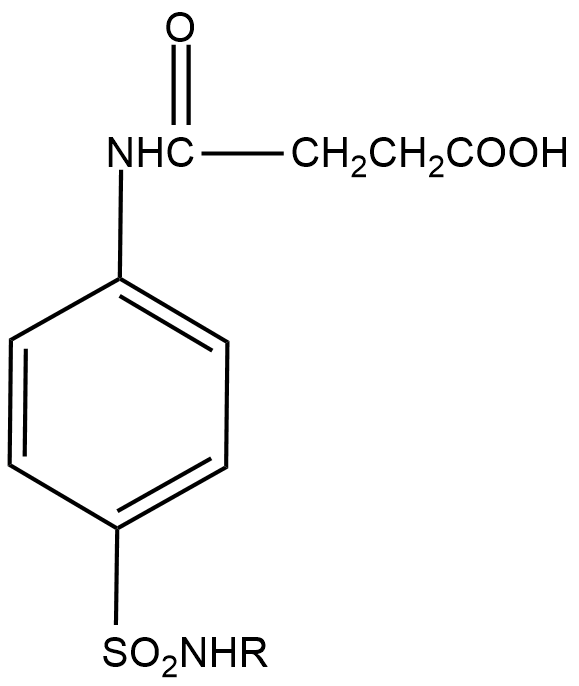 | R=2-pyrimidinyl |
| SA-H | 272.28 |  | R=H |
| BA-PYR | 412.42 | 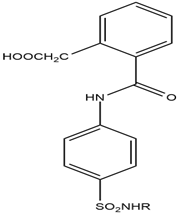 | R=2-pyrimidinyl |
| BA-THIAZ | 417.46 |  | R=2-thiazolyl |
| AA-THIAZ | 353.38 | 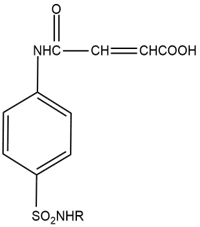 | R=2-thiazolyl |
| PE-H | 334.35 | 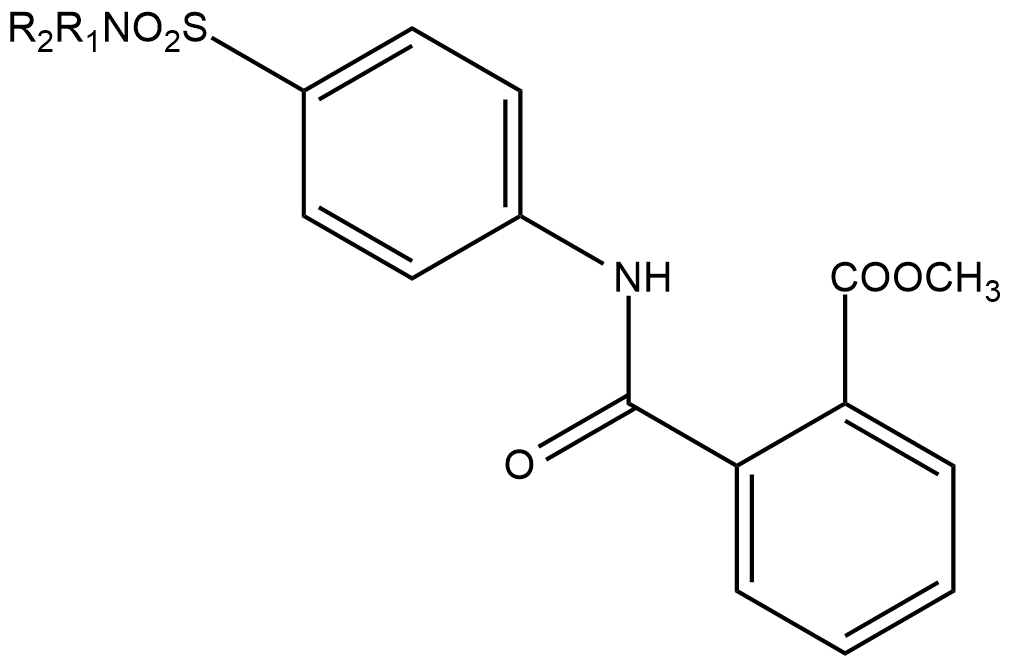 | R1, R2 H, H |
| AE-AMID | 326.33 | 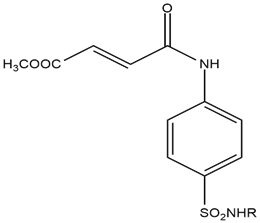 | R1, R2 =C(NH2)2 |

**Table 2S**:

| **Primer** | **Primer sequences** |
| --- | --- |
| CD26 | Forward: GAGATGTTCCGGTCCTGGTCTG  Reverse: TTTGGAGGGCATCTGGACATTC |
| Bcl2 | Forward: 5-TTGTGGCCTTCTTTGAGTTCGGTG -3  Reverse:5- GGTGCCGGTTCAGGTACTCAGTCA-3 |
| GAPDH | Forward: 5-ACAACT TTG GTATCGTGGAAGG-3  Reverse: 5-GCCATCACG CCACAG TTTC-3 |
| VEGF | Forward: 5-CTACCTCCACCATGCCAAGT-3  Reverse: 5-GCAGTAGCTGCGCTGATAGA-3 |

A.

| **Untreated** | **Saxagliptin IC_50_** | **Saxagliptin 0.5 IC_50_** |
| --- | --- | --- |
| 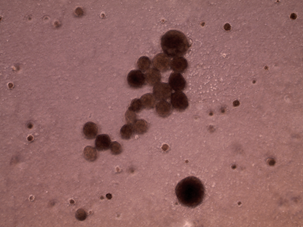4X | 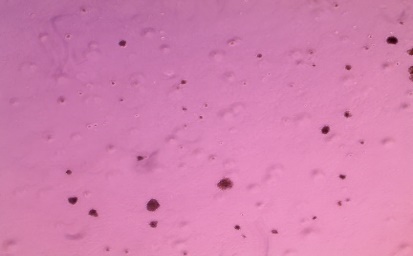 | 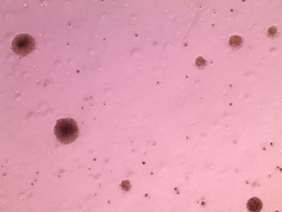 |
| 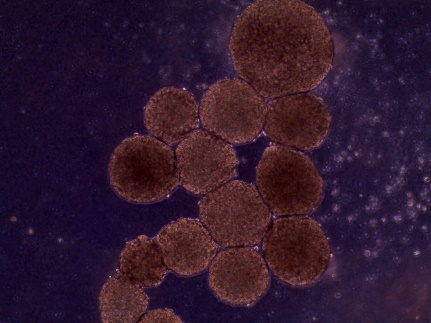10X | 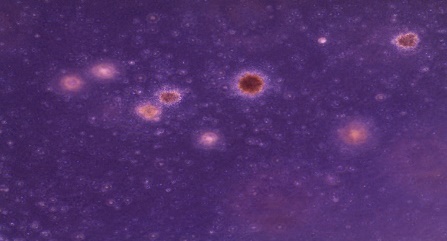 | 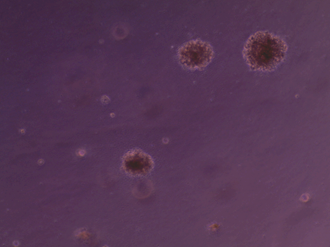 |

B.

| **Untreated** | **AE-AMID IC_50_** | **AE-AMID 0.5 IC_50_** |
| --- | --- | --- |
| 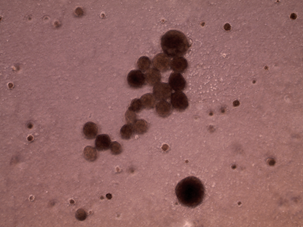4X | 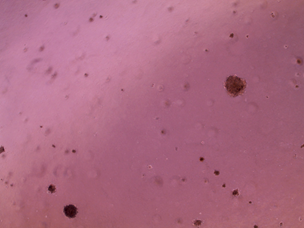 | 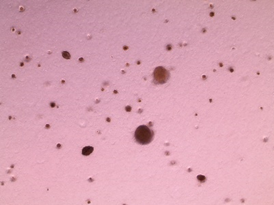 |
| 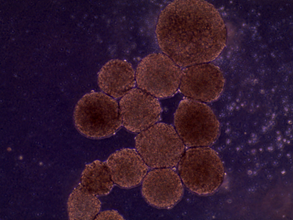10X | 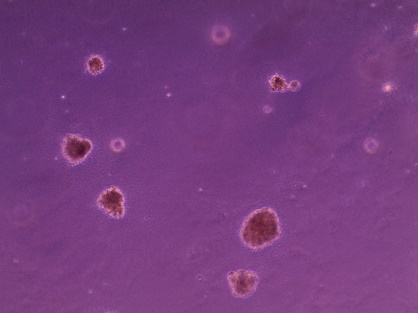 | 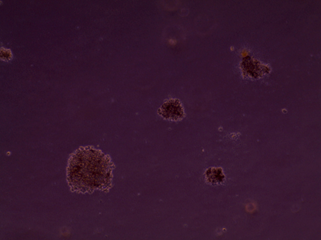 |

C.

| **Untreated** | **Sitagliptin IC_50_** | **Sitagliptin 0.5 IC_50_** |
| --- | --- | --- |
| 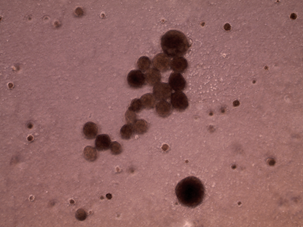4X | 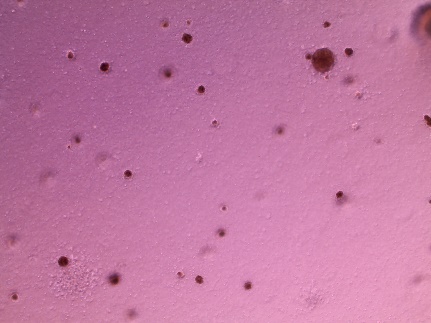 | 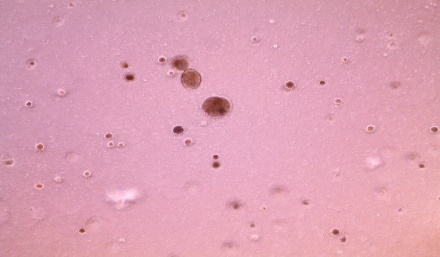 |
| 10X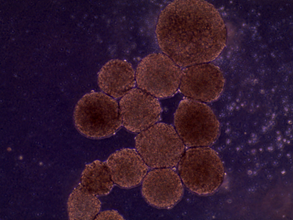 | 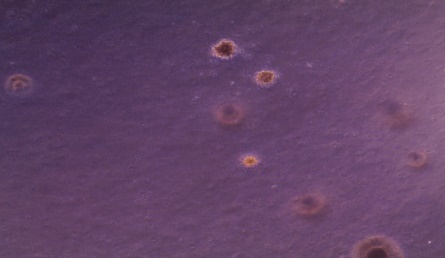 | 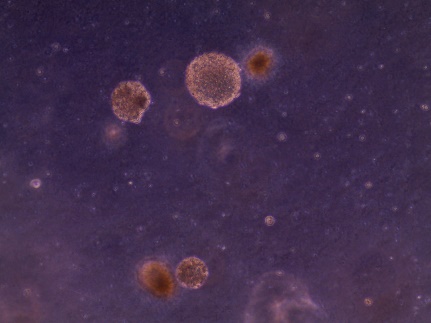 |

D.

| **Untreated** | **PA-AMID IC_50_** | **PA-AMID 0.5 IC_50_** |
| --- | --- | --- |
| 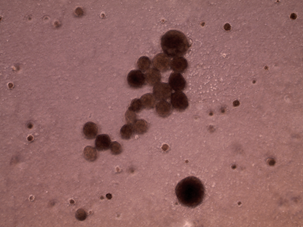4X | 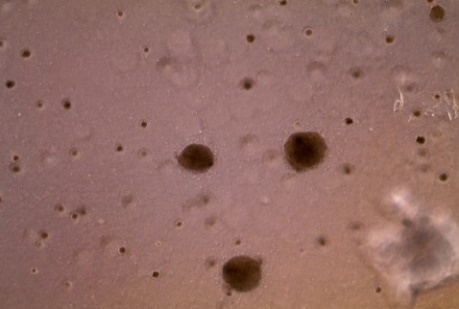 | 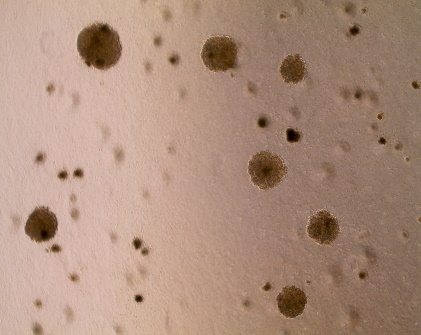 |
| 10X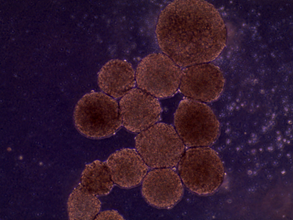 | 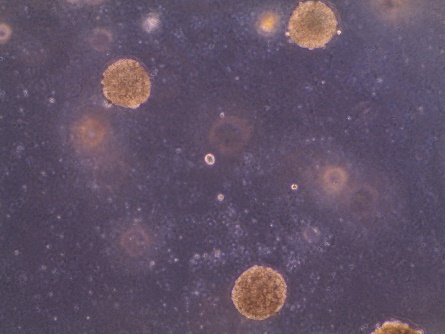 | 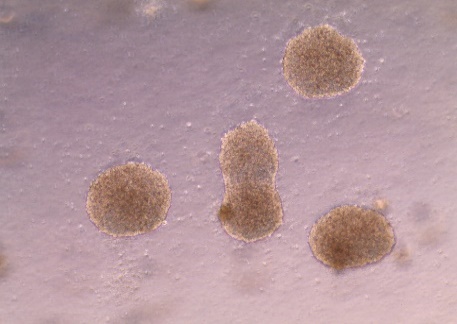 |

Figure 1S. Representative cell colonies in soft agar for HCT116 cells treated with Saxagliptin, Sitagliptin, AE-AMID, and PA-AMID.
